# Deciphering the interferon gene signature spectrum: association with the clinical heterogeneity of Sjögren’s disease

**DOI:** 10.3389/fimmu.2026.1762625

**Published:** 2026-03-18

**Authors:** Ziyue Luo, Ai Chen, Yue Huang, Kaiyuan Zhang, Muzhi Chen, Xinchang Wang

**Affiliations:** 1 School of Basic Medical Sciences, Zhejiang Chinese Medical University, Hangzhou, Zhejiang, China; 2 Second Clinical Medical College, Zhejiang Chinese Medical University, Hangzhou, Zhejiang, China; 3 Department of Rheumatology, The Second Affiliated Hospital, Zhejiang Chinese Medical University, Hangzhou, Zhejiang, China

**Keywords:** disease activity, heterogeneous, interferon, Sjögren’s disease, transcriptomics

## Abstract

**Purpose:**

This study aimed to identify interferon (IFN)-related key genes in patients with Sjögren’s Disease (SjD) and to elucidate their specific associations with the heterogeneous clinical phenotypes and laboratory parameters of the disease.

**Methods:**

Bioinformatics analyses, including differentially expressed gene (DEG) screening, weighted gene co-expression network analysis (WGCNA), and machine learning, were conducted on dataset GSE84844 to identify IFN-related key genes. Based on the EULAR Sjögren’s syndrome disease activity index (ESSDAI), SjD patients with low, moderate, and high disease activity were enrolled, with 20 in each subgroup. Quantitative real-time polymerase chain reaction (qRT-PCR) was performed on peripheral blood mononuclear cells (PBMCs) from the 60 SjD patients and 15 healthy controls (HCs). Expression levels were compared between SjD patients and HCs, across disease activity subgroups, and correlated with clinical phenotypes and laboratory indicators.

**Results:**

DEGs in SjD were significantly enriched in IFN-related signaling pathways. Five IFN-related hub genes were identified: CXCL10, DDX60L, IFIH1, JAK2, and NMI. qRT-PCR validation confirmed that all five genes were significantly upregulated in SjD patients compared to HCs (*P* < 0.05). However, their expression did not significantly differ among SjD subgroups with varying levels of overall disease activity (*P* > 0.05). Importantly, these genes were differentially expressed in distinct clinical manifestations. For instance, elevated CXCL10 expression was observed in patients with interstitial lung disease (ILD), leukopenia, and anemia; JAK2 expression differed in rheumatoid arthritis comorbidity; IFIH1 expression also showed differences in those with Raynaud’s phenomenon and ILD. Furthermore, certain genes were highly expressed in specific laboratory abnormalities: elevated erythrocyte sedimentation rate with CXCL10 and JAK2; hyperglobulinemia with CXCL10, DDX60L, IFIH1, and JAK2; and elevated immunoglobulin G with CXCL10, DDX60L, and IFIH1 (all *P* < 0.05).

**Conclusion:**

This study identifies five IFN-related key genes (CXCL10, DDX60L, IFIH1, JAK2, and NMI) that are upregulated in SjD. Their expression patterns are not generalized markers of disease activity but are specifically linked to distinct clinical phenotypes and serological abnormalities. These findings provide novel mechanistic insights into the clinical heterogeneity of SjD and highlight CXCL10, JAK2, and IFIH1 as potential biomarkers for specific disease complications and promising candidates for targeted therapeutic strategies.

## 1 Introduction

Sjögren’s Disease (SjD) is an autoimmune disease characterized by chronic inflammation of the exocrine glands, leading to dryness of the oral and ocular mucosa following chronic inflammation of the salivary and lacrimal glands (1, 2). Global epidemiological data indicate that the prevalence of SjD is approximately 0.6%, with rates around 0.77% in the United States and ranging from 0.5% to 2.3% in Europe (3). With its global incidence continuing to rise annually, SjD exhibits remarkable clinical heterogeneity, with potential involvement of multiple organ systems including respiratory, cardiovascular, and haematological systems (4–6), patients may present with a variety of systemic manifestations, including ILD, cardiovascular disease, and kidney disease (7). This complexity poses significant challenges for the diagnosis, management, and treatment of SjD (8, 9). Undeniably, SjD exerts a serious impact on public health, highlighting the urgent need to elucidate the potential mechanisms underlying the pathogenesis of SjD, identify reliable biomarkers and therapeutic targets, thereby providing a solid theoretical foundation for improving its diagnosis and treatment.

However, the exact etiology and pathogenesis of SjD remain incompletely understood. Current evidence suggests that genetic susceptibility, immune dysregulation, environmental factors, and endocrine disorders play significant roles in the initiation and progression of SjD, which collectively contribute to the clinical challenges in its diagnosis and treatment (10). Over the past decades, numerous studies have reported significant upregulation of multiple IFN-related genes in the peripheral blood and target tissues of patients with SjD, a phenomenon commonly referred to as the “IFN signature” (11). Existing evidence suggests that these upregulated IFN-related genes may play a crucial role in the pathogenesis of SjD by activating key biological processes involved in disease progression. Studies have demonstrated that IFN-related factors are closely associated with the progression of SjD’s systemic extra-glandular manifestations, as well as the excessive production of autoantibodies and inflammatory cytokines (12, 13),which varies between patient subgroups, linking distinct clinical phenotypes to immune and metabolic processes (14). Therefore, inhibition of excessive IFN activation holds promise as a potential therapeutic strategy for SjD (15).

**Figure 1:**
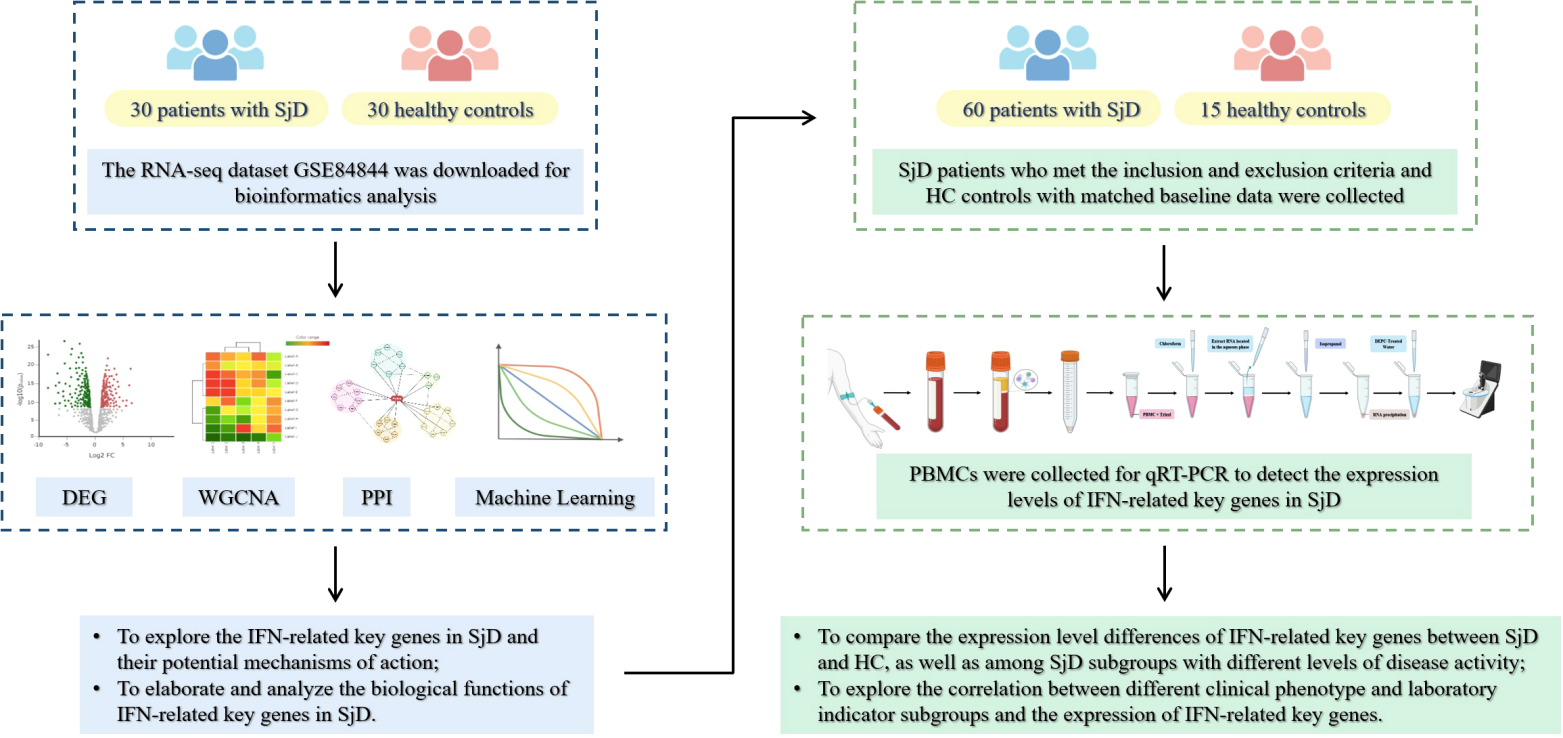
Graphical abstract.

With the increasing advancement of second-generation sequencing technologies and bioinformatics, growing efforts have been devoted to the identification of potential key genes and therapeutic targets in human diseases (16). Numerous studies have utilized bioinformatics analysis to explore hub genes in the pathogenesis of SjD and to validate their potential utility as diagnostic biomarkers in SjD (17). Building on this foundation, the present study integrates transcriptomics and bioinformatics approaches to systematically investigate IFN-related key genes in SjD and elucidate their underlying mechanisms. Moreover, it seeks to delineate potential associations between these IFN-related genes and the heterogeneous clinical phenotypes of SjD. The study flowchart is shown in **Figure 1**.

## 2 Methods

### 2.1 Acquisition of RNA sequencing dataset GSE84844

The RNA-seq dataset GSE84844 was downloaded from the Gene Expression Omnibus (https://www.ncbi.nlm.nih.gov/) using the GEOquery package (v 2.68.0). This dataset comprises peripheral blood RNA-seq data from 30 patients with SjD and 30 healthy controls (HCs).

### 2.2 Identification and enrichment analysis of differentially expressed genes

DEGs were identified in SjD patients vs. HCs using the Limma package (v 3.56.2), with the threshold set at adj.P.Val < 0.05 and |log_2_FC| > 0.5. Heatmaps showing the expression of DEGs were generated using the pheatmap package (v 1.0.12). Enrichment analysis of the DEGs was performed with ReactomePA (v 1.44.0). Furthermore, gene set variation analysis was conducted to calculate the enrichment scores of IFN-related pathways within the DEGs.

### 2.3 Identification of IFN-related key modules and genes

Weighted Gene Co-expression Network Analysis (WGCNA, v1.72.1) was used to perform network modularity analysis on the GSE84844 dataset. Data cleaning was first performed by filtering the top 95% genes ranked by median absolute deviation, followed by sample clustering analysis. Hierarchical clustering was performed using the stats package (v 4.3.1) based on the mean, and outlier samples were removed. Next, a soft threshold of 3 selected to ensure that the constructed network conformed to the characteristics of a scale-free topology. The clustering effect is better when the scale-free topology fitting index is greater than 0.85 and the slope of the change curve changes the most. Module clustering diagrams and module correlation heat maps were then plotted. Based on the screening criteria of correlation coefficients greater than 0.5, key modules related to the IFN-related pathway in SjD were identified. Genes within these modules were further filtered based on correlation coefficients greater than 0.5 with the module to obtain IFN-related genes in SjD. Finally, a Venn diagram was constructed to illustrate the overlap between DEGs and IFN-related genes, defined as IFN-related DEGs.

### 2.4 Enrichment analysis of IFN-related DEGs and construction of protein-protein interaction network

The Search Tool for Recurring Instances of Neighbouring Genes (STRING) database (https://string-db.org/) was employed to perform Gene Ontology (GO), Kyoto Encyclopedia of Genes and Genomes (KEGG), and Reactome enrichment analyses for IFN-related DEGs. The top 10 enriched pathways were visualized using the ggplot2 package. Furthermore, PPI network analysis of IFN-related DEGs was conducted through the Metascape database (https://metascape.org/gp/index.html), from which highly intrinsically correlated functional modules were identified.

### 2.5 Identification and expression verification of IFN-related key genes

Genes with degree > 0 were first extracted from the PPI network diagram and further filtered to distinguish from SjD patients vs. HCs using machine learning approaches. A Random Forest analysis was conducted using the random forest package (v 4.7.1.1). The error rate is basically stable when the number of random trees exceeded 200. With the increasing number of network genes, the error rate fluctuated, and the minimal error rate was observed when the top 13 ranked genes were selected. Subsequently, Least Absolute Shrinkage and Selection Operator (LASSO)-Cox Proportional Hazards Mode (Cox) proportional hazards regression was conducted with the glmnet package (v 4.1.8), in which the lambda.1se (representing the optimal fit for gene screening) was used to identify target genes. Support Vector Machine (SVM)-recursive feature elimination (SVM-RFE) was carried out with the e1071 package (v 1.7.13), achieving the highest accuracy and lowest error rate when the top 38 ranked genes were selected. The intersection of the three machine learning-derived gene sets was defined as IFN-related key genes. Difference between groups was evaluated using Rank sum test, and the expression levels of the IFN-related key genes in the original dataset were visualized with ggplot2.

### 2.6 Immune infiltration analysis of IFN-related key genes

Cibersort analysis was performed on the GSE84844 dataset using the Cibersortx database (https://cibersortx.stanford.edu/) to compare the immune infiltration scores of 22 cell types between groups. Pearson correlation analysis was subsequently performed using the base package (v 4.3.1) to assess the correlation between IFN-related key genes and differential immune cells.

### 2.7 Clinical sample collection and grouping

This study prospectively enrolled SjD patients treated between June 2023 and December 2024 at the Second Affiliated Hospital and the First Affiliated Hospital of Zhejiang Chinese Medical University, as well as the Sir Run Run Shaw Hospital affiliated to Zhejiang University School of Medicine, and the Second Affiliated Hospital of Zhejiang University School of Medicine. The diagnosis of SjD was based on current international criteria (18). Exclusion criteria included active infections, malignancies, acute cardiocerebrovascular diseases, pregnancy or lactation, or any other conditions considered inappropriate for participation by the investigators. Based on the European League Against Rheumatism Sjögren’s Syndrome Disease Activity Index (ESSDAI) (19), patients were categorized into three groups according to disease activity: low (ESSDAI < 5), moderate (5 ≤ ESSDAI ≤ 13), and high (ESSDAI ≥ 14). Under the premise of matching data between groups, 20 SjD patients were included in each group, totaling 60 SjD patients as the disease group. Additionally, 15 baseline-matched HCs were recruited from individuals undergoing health examinations at the Second Affiliated Hospital of Zhejiang Chinese Medical University, and they were defined as the control group.

### 2.8 Ethical approval

This study was conducted in strict compliance with the Declaration of Helsinki and relevant Chinese research guidelines for clinical trials. Prior to initiation, this study was approved by the following listed ethical review committees:

The Second Affiliated Hospital of Zhejiang Chinese Medical University: No. 2023-No. 003-A01; The First Affiliated Hospital of Zhejiang Chinese Medical University: No. 2023-KLS-131-01, No. 2023-KLS-131-02; The Second Hospital Affiliated to Zhejiang University School of Medicine: No. 2023-No. 0490; The Sir Run Run Shaw Hospital affiliated to Zhejiang University School of Medicine: No. 2023-No. 0262.

All participants were fully informed about the study and provided written informed consent prior to enrollment. Participation was voluntary, and all subjects signed the consent forms before the commencement of the study.

### 2.9 Extraction of peripheral blood mononuclear cells

**Figure 2:**
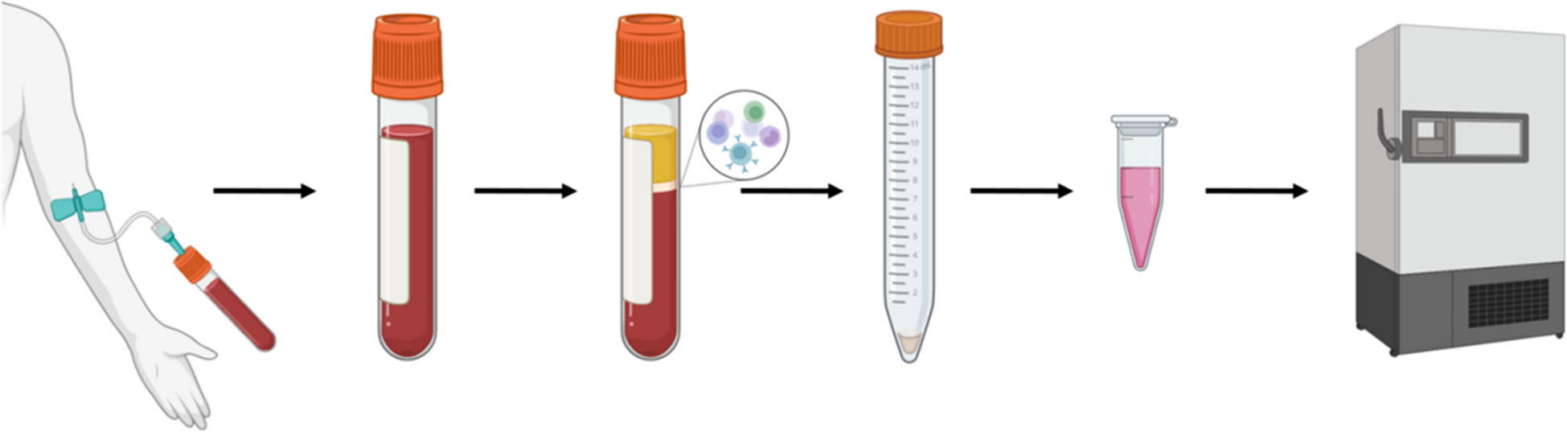
Schematic diagram of PBMC extraction process.

Peripheral blood (4 ml) was collected from each of the 60 SjD patients and 15 HCs into EDTA anticoagulant tubes. Fresh whole blood was transferred into 15 ml centrifuge tubes and diluted 1:1 with phosphate-buffered saline (PBS, 4 ml). The diluted blood was then slowly and carefully layered over 4 ml of lymphocyte isolation buffer in a 15 ml centrifuge tube, ensuring clear interface and avoiding mixing of the two layers. This tube was centrifuged at 2,500 rpm for 20 min, resulting in distinct layers. PBMC layer, appearing as a white interface, was carefully aspirated using a 1000 μl pipette and transferred to a clean 15 ml centrifuge tube. The collected cells were washed with 10 ml PBS. The tube was then centrifuged at 1,500 rpm for 6 min, followed by removal of the supernatant. This washing process was repeated once. Finally, 1ml of Trizol was added and mixed thoroughly, with the mixture temporarily cryopreserved at -80°C ultra-low temperature freezer (**Figure 2**).

### 2.10 Design of primer sequences

The mRNA coding sequences of key genes were obtained from the official website of the National Center for Biotechnology Information (NCBI, https://www.ncbi.nlm.nih.gov/). Specific amplification primers for CXCL10, DDX60L, IFIH1, JAK2, and NMI were designed using the primer design tool provided by Shanghai Sangon Biotech Co., Ltd., and primers were subsequently synthesized by the same company. The primer sequences used for quantitative real-time polymerase chain reaction (qRT-PCR) are detailed in **Table 1**.

**Table 1:** Primer sequences for qRT-PCR.

Gene	Primer	Primer sequence (5 ‘-3’)
CXCL10	CXCL10-F	GAAGGGTGAGAAGAGATGTCTGAATC
	CXCL10-R	TAGACCTTTCCTTGCTAACTGCTTTC
DDX60L	DDX60L-F	GCTACTGAAACACTTGCCTTAGGG
	DDX60L-R	TCTTCCAGCACGACCAGACATC
IFIH1	IFIH1-F	TCCGCTATCTCATCTCGTGCTTC
	IFIH1-R	ACTGTCCTCTGAATCTGCTCCTTC
JAK2	JAK2-F	TGAAGAGCACCTAAGAGACTTTGAAAG
	JAK2-R	TTACGCCGACCAGCACTGTAG
NMI	NMI-F	GGACAGTGCTTCTGACAGGAATG
	NMI-R	TGACCACATCTACTTCTCCACCTC

F, forward primer; R, reverse primer.

### 2.11 RNA extraction

**Figure 3:**
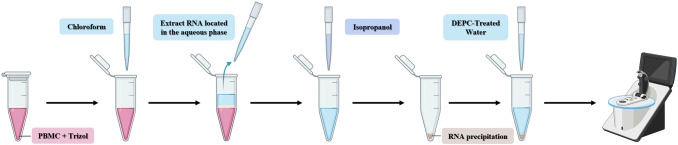
Schematic diagram of RNA extraction process.

The temporarily frozen samples were placed at room temperature for 5 min to ensure completely thawing. Chloroform was added at a ratio of 200 μl per 1 ml of Trizol, followed by vigorous shaking for 1 min and incubation at room temperature for 5 min. Subsequently, centrifugation was performed at 12000 rpm for 15 min at 4°C. 400-500 μl of the supernatant in the colorless transparent layer was carefully pipetted and transferred to a new EP tube. Isopropanol was then added at a ratio of 500 μl per 1 ml of Trizol, mixed by gentle inversion, and incubated at room temperature for 10 min to precipitate RNA. The mixture was centrifuged at 12000 rpm for 10 min at 4°C, and the supernatant was carefully discarded without disturbing the RNA pellet. RNA was washed with 75% ethanol prepared with DEPC-treated water and absolute ethanol, using a ratio of 1 ml 75% ethanol per 1 ml Trizol, mixed gently by inversion, and incubated at room temperature for 5 min to precipitate RNA. Then, centrifugation was performed at 7500 rpm for 5 min at 4°C, and the supernatant was carefully removed. The RNA pellet was dried at room temperature for 5 min until semi-transparent, and then dissolved in 20 μl of DEPC-treated water pre-warmed to 60 °C. RNA concentration was determined using a Nano-Drop spectrophotometer, and the final concentration of the sample was adjusted to 200 ng/ul (**Figure 3**).

### 2.12 qRT-PCR

cDNA was synthesized using the Rapid Reverse Transcription Kit (First-Strand cDNA Synthesis). The resulting cDNA served as the template for qRT-PCR to detect mRNA expression levels. Two replicate wells were set up for each sample. After spotting and labelling on a 96-well plate, the samples were placed in a Light cycler-96 real-time fluorescent quantitative PCR instrument for reaction. Upon completion of the reaction, the PCR results were exported, and the mRNA expression levels of key genes were calculated using the 2^-ΔΔCt^ method, with the human ACTB internal reference primer as the internal reference gene.

### 2.13 Statistical analysis

Statistical analysis and graphical representation of results were performed using SPSS (v 27.0) and GraphPad Prism (v 8.0.2). Data conforming to a normal or approximately normal distribution were presented as mean ± standard deviation, whereas non-normally distributed data were presented as median (upper quartile, lower quartile). For comparisons between two groups, normally distributed data were analyzed using the independent samples t-test while non-normally distributed data were analyzed using the Mann-Whitney U test. For comparisons among multiple groups, normally distributed data were analyzed using one-way analysis of variance, and non-normally distributed data were analyzed using the Kruskal-Wallis H test. Intergroup comparison of categorical data was performed using the chi-square test. *P* < 0.05 indicates a statistically significant difference. *P < 0.05, **P < 0.01, ***P < 0.001, and “ns” indicate non-significant difference.

## 3 Results

### 3.1 Identification of DEGs in SjD

**Figure 4:**
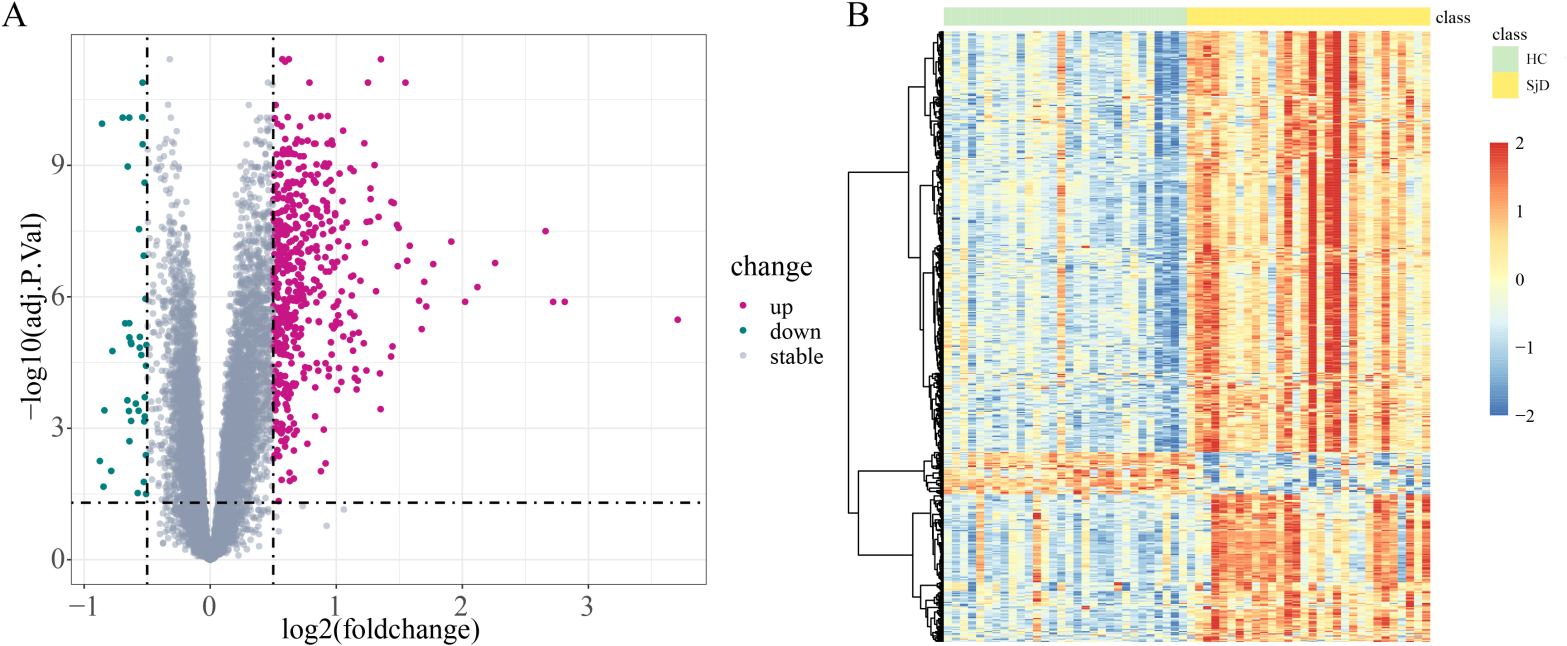
DEGs in SjD patients vs. HCs in the GSE84844 dataset. **(A)** Volcano plot illustrating the DEGs between SjD patients and HCs, with red, green, and gray denoting upregulated genes, downregulated genes, and genes with non-significant differential expression, respectively. **(B)** Heat map showing DEGs between SjD patients and HCs, with light green indicating HC; pale yellow indicating SjD, deeper blue indicating lower gene expression levels, and deeper red indicating higher gene expression levels.

By setting the screening threshold as adj.P.Val < 0.05 and |log_2_FC| > 0.5, a total of 563 DEGs were identified in SjD patients vs. HCs in the GSE84844 dataset, including 524 upregulated and 39 downregulated DEGs. Volcano plots and heat maps were generated to visualize the expression of these DEGs (**Figures 4A, B**).

### 3.2 Enrichment of DEGs in IFN-related pathways in SjD

**Figure 5:**
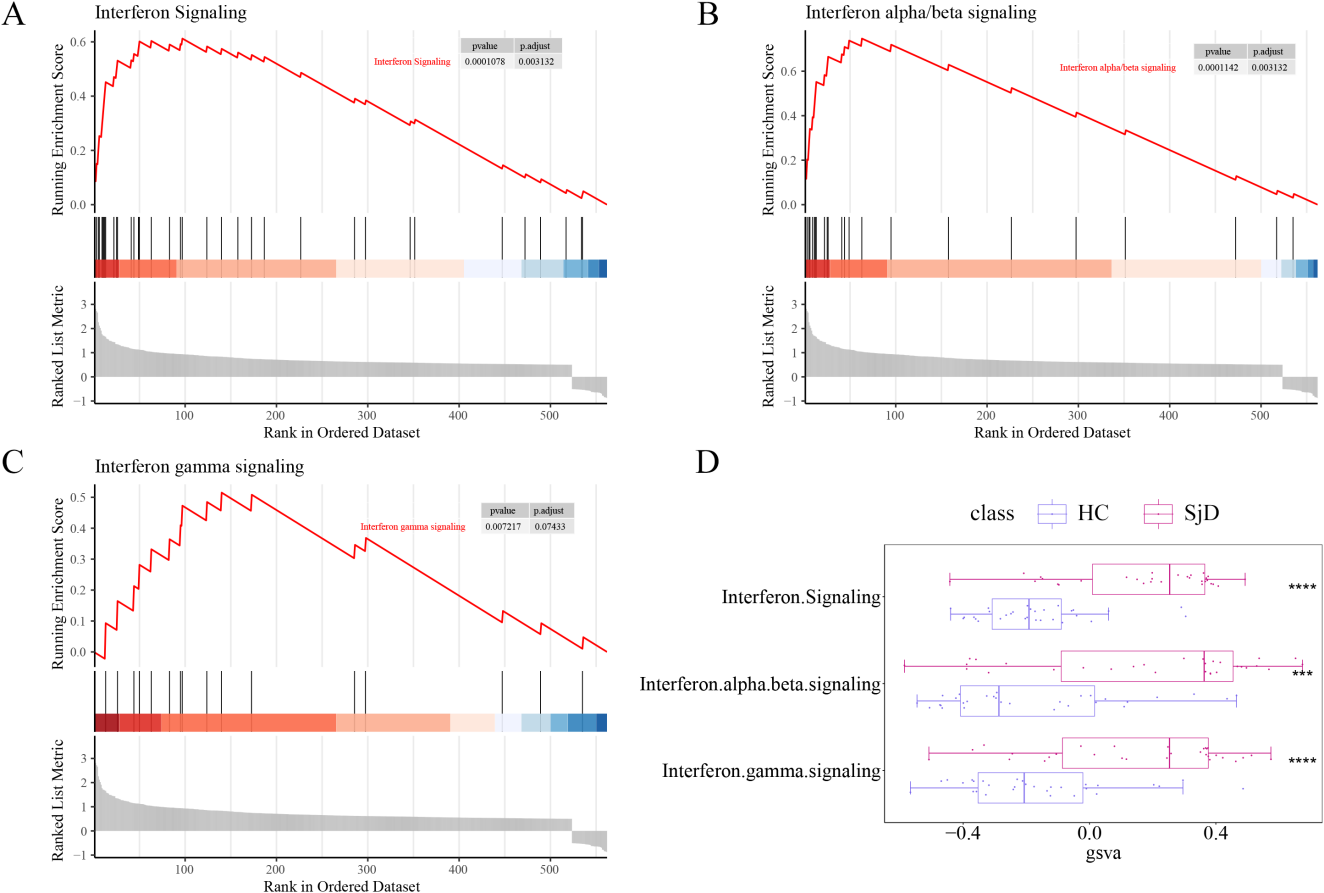
Enrichment of DEGs in IFN-related pathways in the GSE84844 dataset. **(A)** Enrichment of DEGs in the IFN signaling pathway. **(B)** Enrichment of DEGs in the IFN α/β signaling pathway. **(C)** Enrichment of DEGs in the IFN γ signaling pathway. **(D)** Box plots illustrating the expression differences of the three IFN-related pathways between SjD patients and HCs. Note: ***P < 0.001, ****P < 0.0001.

To further investigate the biological functions of the 563 identified DEGs, enrichment analysis were performed. The DEGs were demonstrated to be significantly enriched in three IFN-related pathways: IFN signaling, IFN-α/β signaling, and IFN-γ signaling (**Figures 5A–C**). Additionally, the enrichment scores for these three IFN-related pathways were calculated within DEGs, confirming that all three pathways exhibited significant expression differences in SjD patients vs. HCs (**Figure 5D**).

### 3.3 Identification of IFN-related key modules and genes

**Figure 6:**
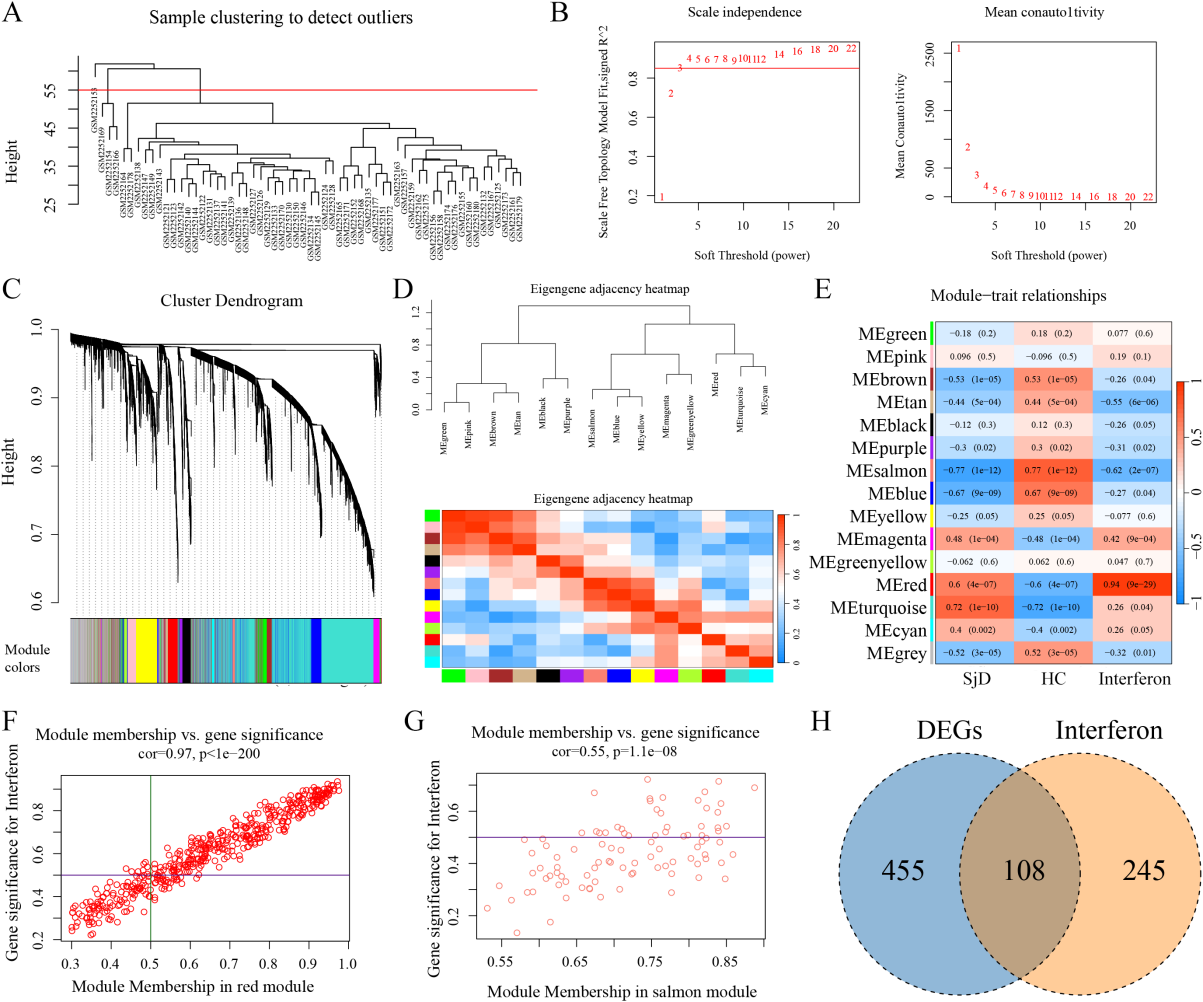
WGCNA clustering results of IFN-related modules in the GSE84844 dataset. **(A)** Cluster results of the top 95% of genes in terms of median absolute deviation. **(B)** Network construction with a soft threshold of 3 is more consistent with the characteristics of scale-free network. **(C)** Gene clustering results corresponding to assigned colors. **(D)** Heatmap illustrating correlations among different modules. **(E)** Heatmap demonstrating correlations between modules and gene sets comprising SjD, HC, and three IFN pathways. **(F)** Scatter plot showing gene distribution and correlations within the red module. **(G)** Scatter plot showing gene distribution and correlations within the salmon module. **(H)** Venn diagram illustrating the overlapping genes between DEGs and IFN-related genes.

Given the significant enrichment of DEGs in IFN-related pathways, we conducted a more in-depth network modularity analysis focusing on IFN within the GSE84844 dataset. First, all genes in the dataset were clustered, and the optimal soft threshold and clustering performance were determined (**Figures 6A–C**). Based on this, a heatmap of the module clustering results was generated (**Figure 6D**), and correlations between different modules and gene sets composed of SjD, HC, and three IFN pathways (**Figure 6E**). Based on the screening criteria of correlation coefficients greater than 0.5, two gene modules, namely the salmon and red modules, were identified. Within these two modules, genes with correlation coefficients greater than 0.5 with both the module were selected, resulting in the identification of 353 IFN-related genes (**Figures 6F, G**). To identify IFN-related DEGs in SjD, the 563 previously identified DEGs were intersected with the 353 IFN-related genes, ultimately yielding a final set of 108 IFN-related DEGs (**Figure 6H**).

### 3.4 Enrichment analysis and PPI network of IFN-related DEGs

**Figure 7:**
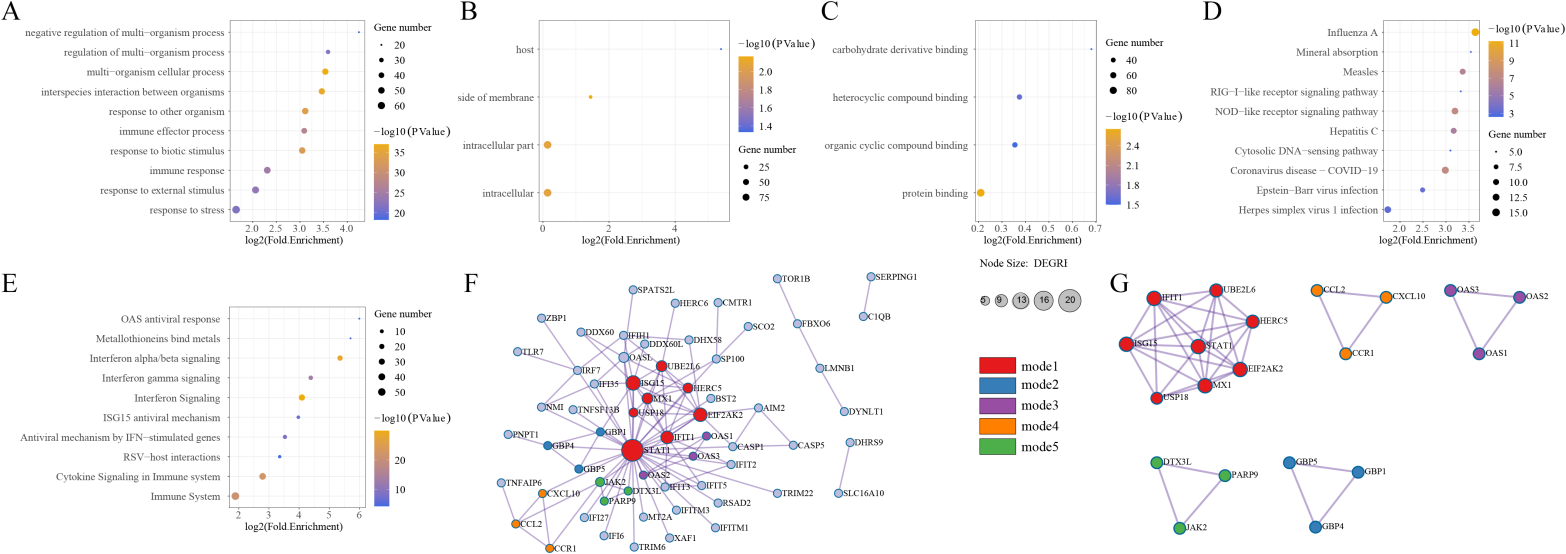
Enrichment analysis and PPI network of IFN-related DEGs in the GSE84844 dataset. **(A)** Dot plot showing the GO-Biological Process (BP) enrichment of IFN-related DEGs. **(B)** Dot plot showing the GO-Cellular Component (CC) enrichment of IFN-related DEGs. **(C)** Dot plot showing the GO-Molecular Function (MF) enrichment of IFN-related DEGs. **(D)** Dot plot showing the KEGG pathway enrichment of IFN-related DEGs. **(E)** Dot plot showing the Reactome pathway enrichment of IFN-related DEGs. **(F)** PPI network of IFN-related DEGs. **(G)** Five highly intrinsically interconnected modules within the PPI network.

Among the 108 IFN-related DEGs, we performed GO, KEGG, Reactome enrichment analyses, as well as PPI analysis. These IFN-related DEGs were shown to be significantly enriched in pathways including immune response, IFN signaling and NOD-like receptor signaling pathway, which, to certain extent, confirmed that IFN-related DEGs may influence immune responses by participating in relevant immuno-inflammatory signaling pathways, thereby playing an important role in the pathogenesis of SjD (**Figures 7A–E**). PPI analysis was conducted to further explore the interaction network among IFN-related DEGs, through which a total of five highly internally correlated modules were obtained within the PPI network (**Figures 7F, G**), indicating that IFN-related DEGs may have close gene-gene interactions that contribute collectively to the pathogenesis of SjD.

### 3.5 Identification of IFN-related key genes via machine learning

**Figure 8:**
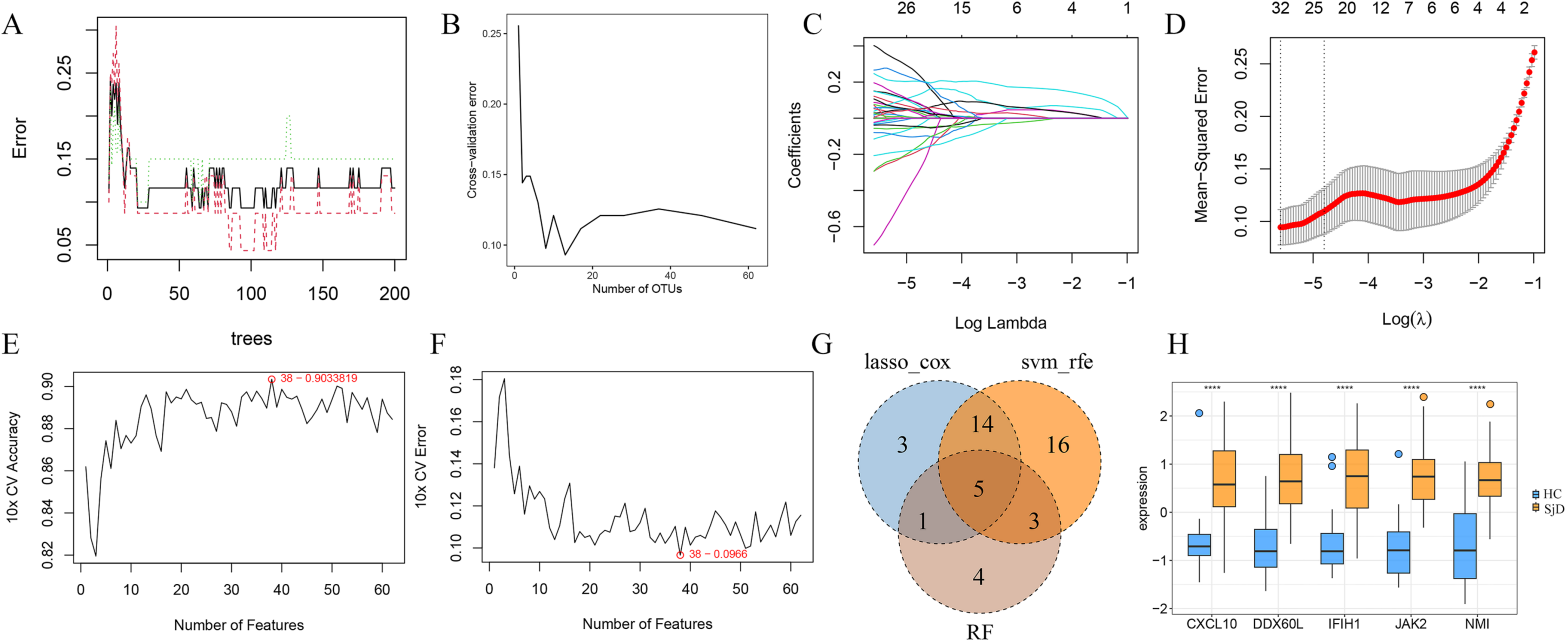
Identification of IFN-related key genes in the GSE84844 dataset via machine learning. **(A)** Error rate corresponding to the number of trees in RF analysis. **(B)** Error rate corresponding to the number of network genes in RF analysis. **(C)** Coefficient distribution of different genes in LASSO-COX analysis. **(D)** Gene selection results under different fitting conditions in LASSO-COX analysis. **(E)** Accuracy corresponding to the number of genes in SVM-RFE analysis. **(F)** Error rate corresponding to the number of genes in SVM-RFE analysis. **(G)** Venn diagram illustrating the intersection of key genes identified by three machine learning methods. **(H)** Box plot demonstrating the expression differences of IFN-related key genes in SjD patients vs. HCs. Note: ****P < 0.0001.

From the PPI network, 62 genes with degree > 0 were selected and subjected to machine learning to further identify IFN-related key genes. A total of 13 (**Figures 8A, B**), 23 (**Figures 8C, D**), and 38 key genes (**Figures 8E, F**) were respectively identified through RF, LASSO-ROX, and SVM-RFE analyses. Through intersection of these machine learning-derived key gene, we identified five IFN-related key genes: CXCL10, DDX60L, IFIH1, JAK2, and NMI (**Figure 8G**). Additionally, expression validation in the original dataset demonstrated that all five IFN-related key genes were significantly upregulated in SjD patients within the GSE84844 dataset (**Figure 8H**).

### 3.6 Investigation of the involvement of IFN-related key genes in the immune microenvironment of SjD

**Figure 9:**
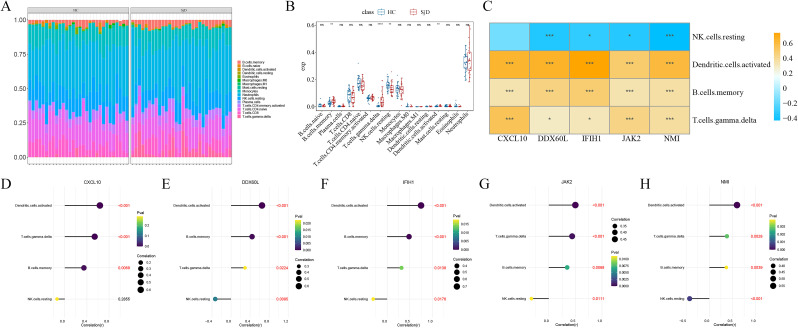
Immune infiltration analysis in the GSE84844 dataset. **(A)** Bar chart showing the proportional distribution of 22 immune cell types across different samples. **(B)** Box plot illustrating the different expression levels of 22 immune cell types between the disease and control groups. **(C)** Heat map showing the correlations between IFN-related key genes and differential immune cells. **(D–H)** Lollipop chart demonstrating the correlation between CXCL10, DDX60L, IFIH1, JAK2, NMI and differential immune cells. Note: *P < 0.05, **P < 0.01, ***P < 0.001.

IFN has been reported to be in close association with the immune microenvironment of SjD. Here, we explored the relationship between IFN-related key genes and the immune microenvironment in SjD. First, immune infiltration analysis was performed on the GSE84844 dataset, and the proportional distribution of 22 types of immune cells in each sample was visualized (**Figure 9A**). Meanwhile, comparison of immune infiltration scores between SjD patients and HCs revealed that four types of immune cells, namely memory B cells, γδ T cells, resting natural killer (NK) cells, and activated dendritic cells (DCs), were significantly differentially expressed between SjD patients and HCs (**Figure 9B**). These results suggest these immune cells may play particularly crucial roles in within the immune microenvironment of SjD. Subsequently, correlation analyses between the five IFN-related key genes and the four differential immune cell types were conducted (**Figure 9C**). The results demonstrated that all five IFN-related key genes exhibited strong correlations with the differential immune cells in SjD (**Figures 9D–H**). This finding further supports the involvement of IFN-related genes in the regulation of the immune microenvironment of SjD, thereby influencing the progression of SjD.

### 3.7 Baseline characteristics of enrolled samples

This study included 60 patients with SjD as the disease group and 15 HCs as the control group. Among the enrolled patients with SjD, 20 had low disease activity (ESSDAI < 5), 20 had moderate disease activity (5 ≤ ESSDAI ≤ 13), and 20 had high disease activity (ESSDAI ≥ 14). Baseline characteristics, including age and sex, were collected and compared between groups. The results showed no significant differences in age or sex between the disease and control groups (*P* > 0.05) (**Table 2**). Furthermore, baseline characteristics were also compared among SjD subgroups with low, moderate, and high disease activity. No significant differences were observed in age, disease duration, or sex across the three subgroups (*P* > 0.05) (**Table 3**).

**Table 2:** Baseline characteristics of SjD patients vs. HCs.

Baseline information	SjD (n=60)	HC (n=15)	*P*
Age (years)	56.50 (49.25, 63.75)	58.00 (46.00, 66.00)	0.853 *^a^*
Sex [male/female (n, %)]	4(6.67%)/56(93.3%)	2(13.3%)/13(86.7%)	0.395 *^b^*

*^a^*is Mann-Whitney U test and *^b^*is Chi-square test.

**Table 3:** Baseline characteristics of SjD patients in across subgroups with different disease activity levels.

Baseline information	Low disease activity (n=20)	Moderate disease activity (n=20)	High disease activity (n=20)	*P*
Age (years)	54.50 (50.75, 61.75)	57.50 (44.25, 67.75)	61.50 (44.25, 64.75)	0.526 *^a^*
Disease duration (years)	5.50 (2.00, 11.75)	5.00 (3.00, 13.50)	6.50 (1.50, 10.75)	0.914 *^a^*
Sex [(male/female (n, %)]	1(5.00%)/19(95.00%)	0(0.00%)/20(100.00%)	3(15.00%)/17(85.00%)	0.153 *^b^*

*^a^*is Kruskal-Wallis H test and *^b^*is Chi-square test.

### 3.8 Expression levels of IFN-related key genes in patients with SjD and HCs

**Figure 10:**
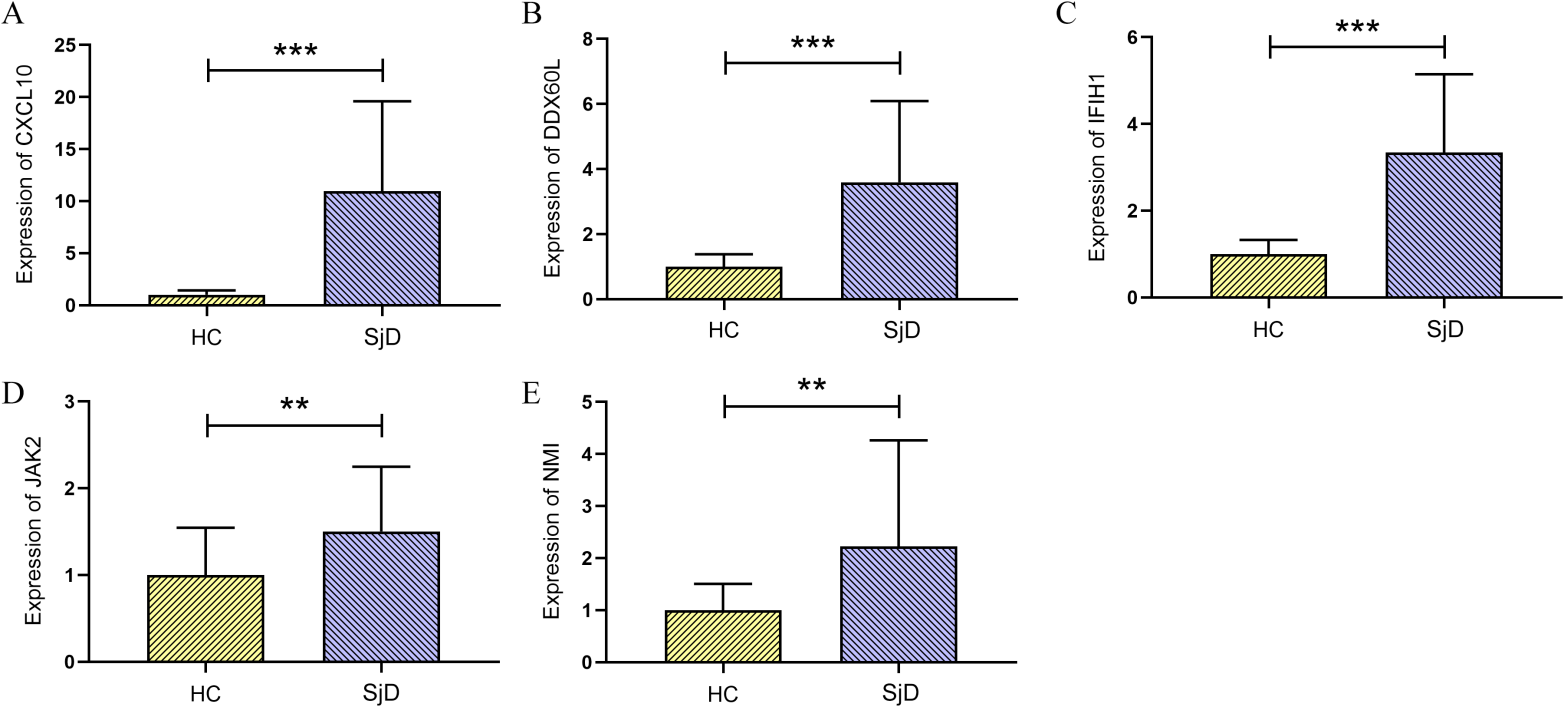
qRT-PCR analysis results of IFN-related key genes in SjD patients vs. HCs. **(A–E)** Bar chart showing mRNA expression levels of CXCL10, DDX60L, IFIH1, JAK2 and NMI. **P < 0.01, ***P < 0.001.

Peripheral blood samples were collected from all enrolled participants for qRT-PCR to assess the expression of the five IFN-related key genes. The results demonstrated that, compared with HCs, patients with SjD exhibited significantly higher expression of CXCL10, DDX60L, IFIH1, JAK2, and NMI (*P* < 0.05), consistent with the findings from previous bioinformatics analysis (**Figures 10A–E**). These findings partially confirm that these five IFN-related key genes are significantly activated during the pathogenesis of SjD and are highly likely to play crucial roles in the pathogenesis and progression of the disease.

### 3.9 Expression levels of IFN-related key genes in SjD patients with different levels of disease activity

**Figure 11:**
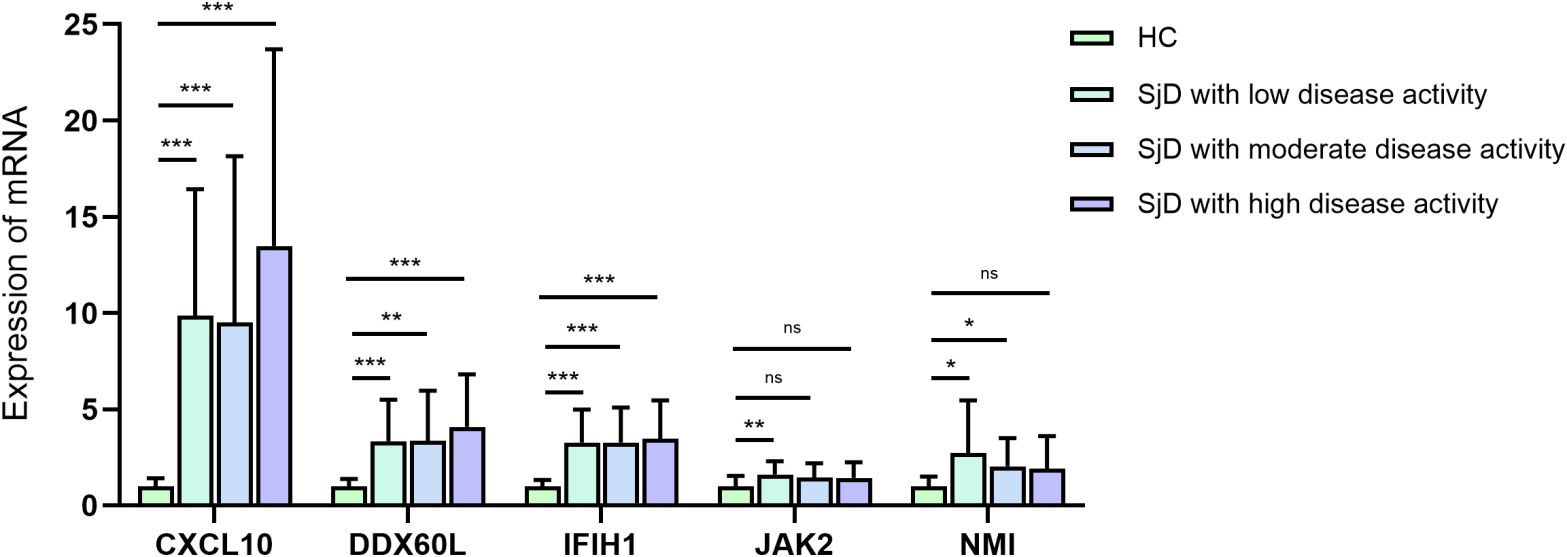
qRT-PCR analysis results of IFN-related key genes in SjD patients with different disease activity and the HCs. *P < 0.05, **P < 0.01, and ***P < 0.001.

To further investigate the expression patterns of IFN-related key genes across SjD patients with varying disease activity, we compared the expression levels of these genes among SjD subgroups with low, moderate, and high disease activity, as well as the control group. The results indicated that CXCL10, DDX60L, IFIH1, JAK2, and NMI were significantly differentially expressed between the three SjD subgroups and the control group (*P* < 0.05) (**Figure 11**). However, no statistically significant differences were observed in the expression levels of these key genes among the three SjD subgroups (*P* > 0.05) (**Table 4**). These findings suggest that the expression of the identified IFN-related key genes may not be strongly associated with disease activity of SjD. It is plausible that that IFN contributes to the pathogenesis of SjD through modulation of other clinical phenotypes rather than directly correlating with disease activity.

**Table 4:** Expression levels of IFN-related key genes across SjD patients with different disease activity.

Group	CXCL10	DDX60L	IFIH1	JAK2	NMI
Low disease activity group (n=20)	9.86 ± 6.59	3.35 ± 2.15	3.28 ± 1.70	1.62 ± 0.68	2.72 ± 2.74
Moderate disease activity group (n=20)	9.52 ± 8.63	3.36 ± 2.61	3.28 ± 1.81	1.45 ± 0.75	2.03 ± 1.47
High disease activity group (n=20)	13.48 ± 10.22	4.09 ± 2.73	3.47 ± 2.00	1.44 ± 0.82	1.92 ± 1.69
*P*	0.270	0.575	0.975	0.368	0.359

### 3.10 Correlation between clinical phenotypes of SjD and IFN-related key genes

To further explore the correlation between each clinical phenotype and IFN-related key genes in SjD, the 60 enrolled SjD patients were stratified into subgroups based on different clinical phenotypes and the expression of IFN-related key genes were compared across these subgroups. The results demonstrated that IFIH1 expression was significantly elevated in SjD patients with Raynaud syndrome compared to those without (*P* < 0.05); CXCL10 and IFIH1 expression were significantly upregulated in SjD patients with interstitial lung disease (ILD) compared to those without (*P* < 0.05); NMI expression was markedly increased in SjD patients with gastroesophageal reflux disease (GERD) versus those without (*P* < 0.05); CXCL10, DDX60L, and IFIH1 expression were significantly elevated in SjD patients with leukopenia compared to those without (*P* < 0.05); CXCL10 expression was significantly higher in SjD patients with anemia compared to those without (*P* < 0.05); CXCL10 and DDX60L expression were markedly increased in SjD patients with thrombocytopenia compared to those without (*P* < 0.05); JAK2 expression was significantly increased in SjD patients with rheumatoid arthritis (RA) compared to those without (*P* < 0.05); CXCL10, DDX60L, and IFIH1 expression were significantly higher in SjD patients with Hashimoto’s disease compared to those without (*P* < 0.05); DDX60L expression was significantly elevated in SjD patients with autoimmune hepatitis versus those without (*P* < 0.05). No significant differences in the expression of IFN-related key genes were observed for other subgroup classifications (*P* > 0.05) (**Tables 5**, **6**). In summary, CXCL10 is differentially expressed in those with ILD, leukopenia, anemia, thrombocytopenia, and Hashimoto’s disease. DDX60L shows differential expression in leukopenia, thrombocytopenia, Hashimoto’s disease, and autoimmune hepatitis. IFIH1 is differentially expressed in Raynaud’s syndrome, ILD, leukopenia, and Hashimoto’s disease. JAK2 expression is altered in RA, while NMI is differentially expressed in GERD.

**Table 5:** Expression levels of IFN-related key genes (CXCL10, DDX60L) in SjD subgroups stratified by clinical phenotypes.

Clinical phenotype		Sample size	CXCL10		DDX60L	
			Expression	*P*	Expression	*P*
Skin	Raynaud syndrome	5	10.91 ± 11.93	0.795	3.02 ± 2.85	0.406
	Vasculitis	4	5.71 ± 5.15	0.278	2.66 ± 1.58	0.450
Musculoskeletal joints	Arthralgia/arthritis	34	11.04 ± 8.33	0.684	3.62 ± 2.45	0.891
	Osteoporosis	10	7.56 ± 6.06	0.211	2.64 ± 2.10	0.135
Kidney	Renal insufficiency	7	7.72 ± 5.45	0.400	2.91 ± 1.15	0.824
Respiratory system	ILD	10	18.14 ± 10.89	***0.013***	3.99 ± 2.97	0.911
Alimentary system	GERD	4	11.87 ± 10.16	0.830	3.46 ± 2.32	0.879
Hematological system	Leukopenia	30	14.66 ± 9.45	***0.001***	4.54 ± 2.59	***<0.001***
	Anemia	13	15.66 ± 10.65	***0.040***	4.51 ± 2.84	0.217
	Thrombocytopenia	11	17.69 ± 8.14	***0.004***	6.03 ± 2.52	***<0.001***
History of autoimmune disease	RA	5	13.33 ± 13.17	0.836	4.69 ± 2.95	0.495
	Systemic lupus erythematosus	3	18.02 ± 10.41	0.174	4.74 ± 3.25	0.499
	Hashimoto’s disease	9	17.47 ± 11.10	***0.041***	5.69 ± 2.59	***0.008***
	Autoimmune hepatitis	9	13.05 ± 8.46	0.438	6.14 ± 2.83	***0.002***

Values in bold indicate P < 0.05.

**Table 6:** Expression levels of IFN-related key genes (IFIH1, JAK2, NMI) in SjD subgroups stratified by clinical phenotypes.

Clinical phenotype		Sample size	IFIH1		JAK2		NMI	
			Expression	*P*	Expression	*P*	Expression	*P*
Skin	Raynaud syndrome	5	5.68 ± 1.70	***0.005***	1.38 ± 0.79	0.601	3.29 ± 2.53	0.248
	Vasculitis	4	3.03 ± 1.53	0.920	1.23 ± 0.54	0.540	2.12 ± 0.99	0.742
Musculoskeletal joints	Arthralgia/arthritis	34	3.64 ± 2.01	0.354	1.65 ± 0.84	0.165	2.37 ± 2.35	0.935
	Osteoporosis	10	3.32 ± 2.32	0.463	1.33 ± 0.76	0.320	2.06 ± 1.93	0.718
Kidney	Renal insufficiency	7	2.22 ± 0.33	0.115	1.24 ± 0.61	0.340	1.01 ± 0.35	0.110
Respiratory system	ILD	10	5.39 ± 2.14	***0.001***	1.74 ± 0.97	0.448	2.41 ± 2.07	0.884
Alimentary system	GERD	4	4.44 ± 1.60	0.115	1.77 ± 0.80	0.351	5.34 ± 3.80	***0.010***
Hematological system	Leukopenia	30	3.77 ± 1.76	***0.021***	1.65 ± 0.86	0.284	2.27 ± 1.63	0.094
	Anemia	13	3.26 ± 2.00	0.736	1.52 ± 0.73	0.884	1.77 ± 1.50	0.374
	Thrombocytopenia	11	3.83 ± 2.14	0.534	1.37 ± 0.49	0.847	1.72 ± 1.29	0.364
History of autoimmune disease	RA	5	5.20 ± 2.64	0.052	2.69 ± 0.89	***0.002***	3.21 ± 2.57	0.389
	Systemic lupus erythematosus	3	4.52 ± 1.63	0.185	1.00 ± 0.22	0.198	2.78 ± 0.61	0.153
	Hashimoto’s disease	9	4.87 ± 2.16	***0.033***	1.64 ± 0.98	0.820	2.34 ± 3.32	0.369
	Autoimmune hepatitis	9	2.84 ± 1.25	0.611	1.18 ± 0.46	0.197	2.33 ± 1.73	0.582

Values in bold indicate P < 0.05.

### 3.11 Correlation between common laboratory indicators and IFN-related key genes in SjD

To further investigate the relationship between common laboratory indicators and IFN-related key genes in SjD, the 60 enrolled SjD patients were stratified into subgroups based on abnormal laboratory indicators, and the expression of IFN-related key genes were compared across these subgroups. The results showed that CXCL10 and JAK2 were significantly upregulated in SjD patients with abnormally elevated erythrocyte sedimentation rate (ESR) compared to those with normal ESR (*P* < 0.05); NMI was significantly upregulated in SjD patients with abnormally elevated alanine aminotransferase (ALT) compared to those normal ALT (*P* < 0.05); IFIH1 and NMI were significantly upregulated in SjD patients with abnormally elevated alanine aminotransferase (AST) compared to those with normal AST (*P* < 0.05); CXCL10, DDX60L, IFIH1 and JAK2 were significantly upregulated in SjD patients with abnormally elevated globulin (GLB) compared to those with normal GLB (*P* < 0.05); JAK2 was significantly upregulated in SjD patients with abnormally elevated immunoglobulin A (IgA) compared to those with normal IgA (*P* < 0.05); CXCL10, DDX60L, and IFIH1 were significantly upregulated in SjD patients with abnormally elevated immunoglobulin G (IgG) compared to those with normal IgG (*P* < 0.05); and DDX60L was significantly upregulated in SjD patients with abnormally elevated immunoglobulin M (IgM) compared to those with normal IgM (*P* < 0.05). No significant differences in IFN-related key gene expression were observed for other laboratory indicator-based subgroup classifications (*P* > 0.05). Additionally, expression levels of the above genes did not significantly differ between patients positive for ANA (with anti-SSA+, anti-SSB+, or anti-Ro52+) and those who were ANA-negative (*P* > 0.05) (**Tables 7**, **8**). In summary, CXCL10 expression is upregulated in the presence of elevated ESR, GLB, and IgG; DDX60L expression is increased in the presence of elevated GLB, IgG, and IgM; IFIH1 expression is elevated alongside increased AST, GLB, and IgG; JAK2 expression is upregulated in the context of elevated ESR, GLB, and IgA; and NMI expression is increased when ALT and AST are elevated.

**Table 7:** Expression levels of IFN-related key genes (CXCL10, DDX60L) in the SjD subgroups stratified by laboratory indicators.

Clinical phenotype		Sample size	CXCL10		DDX60L	
			Expression	*P*	Expression	*P*
Erythrocyte sedimentation rate	ESR↑	50	12.15±8.92	***0.016***	3.86±2.63	0.163
Biochemistry	CRP↑	10	11.13±10.04	0.920	2.48±2.22	0.074
	ALT↑	10	6.98±6.16	0.096	3.87±2.68	0.609
	AST↑	13	12.14±11.93	0.864	3.70±2.58	0.832
	ALB↓	34	10.68±7.92	0.802	3.49±2.68	0.316
	GLB↑	44	12.65±8.89	***0.006***	4.15±2.55	***<0.001***
	GGT↑	12	11.55±9.39	0.919	4.91±2.96	0.091
Rheumatoid factors	RF↑	26	10.37±7.52	0.530	3.25±2.36	0.617
Immunologic function	IgA↑	28	11.47±8.78	0.575	4.15±2.59	0.064
	IgG↑	38	12.08±7.79	***0.016***	4.11±2.46	***0.002***
	IgM↑	6	15.77±11.23	0.214	5.47±2.38	***0.033***
	C3↓	29	11.76±8.97	0.511	4.01±2.46	0.058
	C4↓	13	11.33±10.45	0.814	3.45±2.28	0.731
Antinuclear Antibody (ANA)	Anti-SSA+	48	10.67±8.56	0.380	3.42±2.40	0.177
	Anti-SSB+	35	11.08±8.78	0.914	3.67±2.46	0.677
	Anti-Ro52+	45	11.85±9.05	0.100	3.83±2.53	0.145

Values in bold indicate P < 0.05.

**Table 8:** Expression levels of IFN-related key genes (IFIH1, JAK2, NMI) in the SjD subgroups stratified by laboratory indicators.

Clinical phenotype		Sample size	IFIH1		JAK2		NMI	
			Expression	*P*	Expression	*P*	Expression	*P*
Erythrocyte sedimentation rate	ESR↑	50	3.44±1.89	0.699	1.61±0.76	***0.017***	2.23±2.14	0.682
Biochemistry	CRP↑	10	3.13±2.13	0.351	1.35±0.56	0.565	1.82±1.67	0.413
	ALT↑	10	3.78±2.18	0.530	1.47±0.59	0.821	4.15±3.15	***0.010***
	AST↑	13	4.56±2.14	***0.013***	1.66±0.87	0.477	2.05±2.07	***0.032***
	ALB↓	34	2.98±1.66	0.058	1.48±0.73	0.752	1.87±1.43	0.632
	GLB↑	44	3.56±1.80	***0.040***	1.62±0.77	***0.047***	2.23±2.14	0.575
	GGT↑	12	3.19±1.94	0.773	1.32±0.62	0.419	3.05±3.01	0.174
Rheumatoid factors	RF↑	26	2.81±1.61	0.080	1.60±0.89	0.848	1.64±1.04	0.654
Immunologic function	IgA↑	28	3.40±1.72	0.690	1.76±0.77	***0.020***	2.29±2.41	0.751
	IgG↑	38	3.71±1.85	***0.029***	1.58±0.78	0.630	2.43±2.24	0.256
	IgM↑	6	3.87±2.07	0.316	1.83±0.70	0.256	2.47±2.04	0.708
	C3↓	29	3.32±1.78	0.981	1.60±0.71	0.321	2.20±1.97	0.782
	C4↓	13	3.67±2.07	0.785	1.64±0.68	0.332	1.85±1.38	0.566
Antinuclear Antibody (ANA)	Anti-SSA+	48	3.24±1.68	0.109	1.57±0.78	0.883	2.24±2.09	0.442
	Anti-SSB+	35	3.29±1.73	0.603	1.54±0.82	0.667	2.36±2.14	0.943
	Anti-Ro52+	45	3.63±1.90	0.059	1.62±0.77	0.181	2.46±2.26	0.397

Values in bold indicate P < 0.05.

## 4 Discussion

To investigate the mechanistic roles of interferon-related key genes in SjD and further elucidate their potential associations with the heterogeneous clinical phenotypes of the disease, the present study explored the “IFN signature” in SjD at the transcriptional level. Specifically, the GSE84844 dataset was selected for bioinformatics analysis. DEG analysis revealed that the DEGs were significantly enriched in IFN-related pathways, including the IFN-α/β signaling pathway and the IFN-γ signaling pathway. Notably, IFN-α and IFN-β, as the most extensively studied IFN-I subtypes, and IFN-γ, the sole member of IFN-II subtype, collectively underscore the crucial role of IFN in the pathogenesis of SjD (20, 21).

To further analyze IFN-related key genes in SjD, PPI analysis and machine learning methods were employed in this study, and five IFN-related key genes (CXCL10, DDX60L, IFIH1, JAK2, and NMI) were found to be significantly upregulated in SjD. Notably, CXCL10 is a small molecule cytokine from the CXC chemokine family produced by multiple cell types upon IFN-γ stimulation. It plays a crucial regulatory role in various immune responses through regulation of T-cell migration (22). In SjD, elevated CXCL10 expression has been observed in salivary glands of SjD patients, and interactions between ligand CXCL10 and the endogenous receptor CXCR3 have been shown to promote inflammation and contribute to the progression of SjD-like xerostomia in NOD (23). It was also speculated that overexpression of CXCL10 in the salivary glands of patients with SjD is primarily driven by IFN-γ-stimulated ductal cells, with IFN-γ-induced upregulation of CXCL10 in these cells likely being the primary cause of inflammatory lesions in the salivary glands of patients with SjD (24). DDX60L is an Adenosine Triphosphate (ATP)-dependent helicase gene involved in IFN-mediated antiviral responses of innate immunity (25). Although the relationship between DDX60L and IFN in SjD remain underexplored, previous studies have demonstrated close associations between DDX60L and various autoimmune diseases, including SLE, autoimmune hepatitis, Behcet’s disease, and multiple sclerosis (MS) (26, 27). In SLE, the significantly high expression of DDX60L is thought to be linked to IFN-mediated innate immune responses and immune activation (26). IFIH1 has also been extensively studied in the context of IFN. Gain-of-function in IFIH1 lead to upregulation of IFN- I responses, and individuals carrying such mutations often exhibit autoimmune disease-like phenotypes, including SLE, dermatomyositis, and ILD (28, 29). Additionally, comparative analysis of IFN signature-positive and -negative SjD patients revealed that IFIH1 expression was specifically elevated only in the presence of IFN signature, whereas in IFN signature-negative SjD patients, IFIH1 expression showed a downward trend (30). This suggests that IFIH1 may serve as an external marker of IFN signature in SjD and potentially holds significant potential as a biomarker for distinguishing different IFN subtypes. JAK2, belonging to the non-receptor tyrosine kinase family, is one of the downstream factors of IFN-γ (31). As a key component of the JAK-STAT signaling pathway, JAK2 is involved in regulating cell proliferation, differentiation, apoptosis, and immune responses. Current evidence supports the critical role of IFN-mediated JAK-STAT signaling in the onset and progression of SjD (32). Interestingly, the JAK1/2 inhibitor Baricitinib has been shown to suppress IFN-γ-induced CXCL10 expression by inhibiting JAK-STAT signaling, thereby reducing immune cell chemotaxis and ultimately ameliorating the disease progression of SjD (33). NMI is another downstream factor induced by IFN. Although its role in the pathogenesis of SjD has not yet been reported, studies in viral infections have demonstrated that NMI interacts with IFN-regulated factors and participate in regulating innate immune processes (34). These findings collectively highlight the critical role of IFN-related key genes in the pathogenesis of SjD, providing novel therapeutic targets and scientific foundations for IFN-based treatments in SjD.

Dysregulation of the immune microenvironment is also a critical aspect of the pathogenesis of SjD, yet the precise role of IFN in this context remains incompletely understood. In this study, after identifying five IFN-related key genes in SjD, we further investigated the biological characteristics of IFN within the immune microenvironment of SjD. Immune infiltration analysis revealed that levels of four immune cells (memory B cells, γδ T cells, resting NK cells, and activated DCs) in SjD patients were significantly elevated in SjD, and all five IFN-related key genes showed high expression variability in the four immune cell populations. Therefore, we reasonably hypothesize that IFN-related key genes may participate in regulating the immune microenvironment, thereby influencing the progression of SjD. This hypothesis is supported by accumulating evidence in the literature. For instance, IFN-I have been shown to induce epigenetic alterations in memory B cell subsets during chronic viral infections (35). Moreover, compared to ANA-negative patients, ANA-positive patients exhibit higher expression of memory B cell subsets following IFN-α stimulation (36). γδ T cells, as an early source cell secreting IFN-γ and tumor necrosis factor-α (TNF-α), have been demonstrated to mediate immune responses through antibody-dependent cytotoxicity targeting tumor cells and indirectly modulate the tumor immune microenvironment via interactions with B cells, NK cells, and DCs (37). Furthermore, activated plasma cell-like DCs, which serve as the most effective producers of IFN- I , can also be induced by IFN-γ to generate large amounts of BAFF, which further accelerate B cell maturation and proliferation, ultimately facilitating autoantibody production and causing severe imbalance in the immune microenvironment of SjD (38). Therefore, modulating the IFN-mediated immune microenvironment stability may become a potential therapeutic approach for future interventions of SjD.

SjD is one of the autoimmune diseases with the most diverse phenotypes, 30-40% of patients with SjD experience more extensive systemic involvement, which significantly increase mortality risks (39). Even among diagnostically relevant autoantibodies, not all SjD patients test positive; for instance, the positivity rate of anti-SSA antibodies ranges from 50-70%, whereas that of anti-SSB antibodies is approximately 30-50%. These findings underscore both the clinical heterogeneity of SjD and the difficulties associated with its clinical diagnosis and treatment, highlighting the necessity for personalized treatment as the mainstream intervention method for SjD patients in the future (40). proteomic network analysis has been employed to explore potential pathological mechanisms underlying the heterogeneity of SjD (41). Proteomic investigations have revealed striking differences in serum protein networks across different SjD subtypes. Certain subtypes demonstrate high expression of transcription factors associated with innate immunity, inflammation, redox balance, and energy metabolism, whereas others exhibit high expression of proteins related to chemokines, cytokines, and lymphocyte activation. These findings collectively indicate that different SjD subtypes are driven by different pathological mechanisms (41).

Based on the current understanding, this study further classified SjD patients into distinct disease subgroups according to their clinical phenotypes and compared the expression of IFN-related key genes among these subgroups. Our analysis revealed that CXCL10 is differentially expressed in ILD, leukopenia, anemia, thrombocytopenia, and Hashimoto’s disease; DDX60L is differentially expressed in leukopenia, thrombocytopenia, Hashimoto’s disease, and autoimmune hepatitis; IFIH1 is differentially expressed in Raynaud’s syndrome, ILD, leukopenia, and Hashimoto’s disease; JAK2 is differentially expressed in RA; and NMI is differentially expressed in GERD. Previous studies have reported that CXCL10 may be potentially related to early-stage ILD in MS (42), and its predictive value as a diagnostic biomarker for RA-associated ILD has been confirmed (43). Our findings highlight significant differences in CXCL10 expression between SjD patients with and without ILD, potentially providing novel theoretical foundations for personalized treatment for SjD patients combined with ILD. Moreover, JAK inhibitors are commonly used in RA management (44), and our findings of significantly elevated JAK2 levels in SjD patients combined with RA align with current RA research, suggesting that JAK2 inhibitors may also hold promising therapeutic potential for this SjD-RA subgroup. Additionally, genetic polymorphisms in IFIH1 and CXCL10 have been reported to be significantly associated with the risk of Hashimoto’s disease, suggesting potential shared genetic mechanisms between SjD patients combined with Hashimoto’s disease and individuals with isolated Hashimoto’s disease (45, 46). Additionally, correlation analysis of SjD subgroups stratified by laboratory parameters demonstrated significant clinical implications. For instance, CXCL10 expression was revealed to be positively correlated with IgG levels. Previous research indicates that upregulation of CXCL10 in various autoimmune diseases often reflects severe immune-inflammatory dysregulation, which further promotes B-cell proliferation and IgG production, and is strongly associated with increased IFN-γ secretion (47). At the level of mechanisms linking genes and phenotypes, the key genes identified in this study may mediate clinical heterogeneity by modulating downstream signaling pathways or intercellular interactions. For example, CXCL10 recruits Th1 cells and monocytes to target organs (such as the lungs and thyroid) through the CXCR3 axis, which can directly promote local inflammation and tissue damage. This may represent the core mechanism underlying its association with phenotypes such as ILD, leukopenia, and Hashimoto’s thyroiditis (48, 49). Similarly, as a core kinase of the JAK-STAT pathway, upregulation of JAK2 can amplify the signaling of multiple cytokines (including IFN-γ and IL-6), thereby driving synovial inflammation and autoantibody production associated with RA (50). This provides a molecular explanation for the pathogenesis of the SjD-RA subgroup. These findings collectively highlight the critical role of IFN-related key genes in the pathogenesis of SjD, providing novel therapeutic targets and scientific foundations for IFN-based treatments in SjD.

However, this study also presents several limitations that warrant further exploration and validation in future research. For instance, due to financial and geographic constraints, the present study was limited to transcriptome-based datasets, and the clinical phenotypes included in the subgroup analysis remain insufficiently comprehensive. Also, the experimental validation confirms upregulation of the selected genes in SjD compared to HCs, but does not validate associations with specific clinical phenotypes. Therefore, the reliability and scientificity of these findings require further validation through larger-scale multicenter clinical cohorts. Moreover, while this study focused solely on identifying IFN-related key gene targets in SjD, subsequent research should incorporate animal or cell experiments to further elucidate the underlying IFN-mediated mechanisms in SjD and to fully explore the therapeutic potential of these IFN-related key genes in guiding personalized treatment strategies.

## 5 Conclusion

The DEGs in SjD were found to be significantly enriched in IFN-related pathways, and the five IFN-related key genes (CXCL10, DDX60L, IFIH1, JAK2, and NMI) were ultimately identified as closely associated with the immune microenvironment of SjD. Furthermore, all these five key genes were significantly upregulated in SjD. Although their expression did not differ significantly across SjD subgroups with varying disease activity, significant differences in the expression of these genes were observed among SjD patients in subgroups stratified by different clinical phenotypes and laboratory parameters.

## Data Availability

The datasets presented in this study can be found in online repositories. The names of the repository/repositories and accession number(s) can be found in the article/supplementary material.
